# A protozoan perspective on climate change and biosafety threats: differences in testate amoebae in lakes in forest–swamp and forest–steppe zones in Western Siberia

**DOI:** 10.1128/aem.00330-25

**Published:** 2025-05-14

**Authors:** Olga N. Zagumyonnaya, Dmitry G. Zagumyonnyi, Elena A. Gerasimova, Denis V. Tikhonenkov

**Affiliations:** 1 AquaBioSafe Laboratory, University of Tyumen, Tyumen, Russia; 2 Papanin Institute for Biology of Inland Waters, Russian Academy of Sciences 133852 , Borok, Russia; Colorado School of Mines, Golden, Colorado, USA

**Keywords:** testate amoebae, 18S rRNA gene, protist, plankton communities, high-throughput sequencing, light microscopy, Cercozoa, climate change, biosafety

## Abstract

The problem of increasing salinity and mineralization in natural and artificial freshwater bodies with climate warming is very relevant nowadays, as it leads to changes in the species composition of planktonic organisms. Testate amoebae are one of the responsive bioindicators that are sensitive to even minor changes in environmental conditions. In this study, a comparative analysis of the species diversity of planktonic testate amoebae was carried out in a number of lakes in the forest–steppe and forest–swamp natural zones of Western Siberia using microscopy and metabarcoding. One new species, *Pseudodifflugia siemensmai* sp. nov., was described. The detection frequency and the number of reads of amplicon sequence variants of potentially pathogenic testate amoebae belonging to the genera *Rhogostoma* and *Fisculla* were higher in forest–steppe lakes. Universal eukaryotic primers for the 18S rRNA gene are well suited for identifying testate amoebae from the supergroup Cercozoa but are practically not applicable for identifying Amoebozoa testaceans. The plankton of the lakes with the highest mineralization and salinity was characterized by the most specific species composition. These results should be taken into account when predicting changes in aquatic communities with further climate warming, which may also be associated with an increase in the occurrence of pathogenic testaceans that pose biosafety threats.

**IMPORTANCE:**

Microscopic and metabarcoding analyses reveal important differences in testate amoebae communities in lakes in two natural and climatic zones of Western Siberia that should be taken into account when predicting changes in aquatic communities with further climate warming, which may also be associated with an increase in the occurrence of pathogenic testaceans that pose biosafety threats.

## INTRODUCTION

Aquatic ecosystems of Western Siberia are important model objects of research on a planetary scale. This territory, which includes six natural zones, from arctic deserts to forest–steppes, is convenient for conducting research related to assessing the state and trends in the transformation of aquatic ecosystems and aquatic communities in the context of global changes. Currently, research that can predict changes in the species composition of free-living and pathogenic planktonic organisms during climate warming and that relates to issues of biosafety and aquaculture is very relevant. Testate amoebae, which also include pathogenic species, are good bioindicators (1–3) due to their high sensitivity to minor changes in environmental factors (3–5). However, planktonic testate amoebae are the least studied compared to benthic, sphagnobiont, and soil testaceans. Comparative studies of their communities in lakes of different natural zones with different climatic parameters have not yet been carried out. At the same time, the analysis of testate amoebae in natural samples is quite labor intensive, and the problem of optimizing methods for their study is topical.

Currently, ecological, bioindication, and palaeoecological studies of testate amoebae are based on the morphological identification of species due to the unique structure of the shells of each species of these protists (2, 6–8). Taxonomic analysis based on light and electron microscopy is the main method for studying the species composition of testate amoebae but is time consuming. Modern molecular studies of testate amoebae are making adjustments to the process of identifying testate amoebae, accelerating this process and revealing the hidden diversity of these protists. DNA metabarcoding reveals a large number of new lineages and expands the range of phylogenetic diversity of protists at almost every taxonomic level (9–13). A few studies of testate amoebae diversity using the metabarcoding approach have focused on soil (14–16) and *Sphagnum* bog (17) biocenoses, whereas the amplicon diversity of planktonic testate amoebae of water bodies has not been studied.

The controversial issues of using only one of the methods for determining the taxonomic composition of protists are becoming increasingly significant. Detailed studies are being conducted to compare microscopic and metabarcoding identification for freshwater planktonic ciliates (18, 19), diatoms (20), and pico-, nano-, and microplankton (13, 21–24). However, similar work has not been carried out for testate amoebae.

The development of new approaches to the use of protist metabarcoding in applied ecological studies seems to be very relevant. The DNA metabarcoding is applicable for the identification of bioindicator species of diatoms and foraminifera (11, 25), ciliates (26–28), and testate amoebae within the Amoebozoa supergroup (29) and can also be used to reveal the hidden diversity of pathogenic testate amoebae species of the genus *Rhogostoma* Belar, 1921, from soils (15).

Here we report the results of a microscopic and metabarcoding study and comparative analysis of free-living and pathogenic planktonic testate amoebae from lakes differing in mineralization and other hydrochemical characteristics, located in different climatic zones of Western Siberia within the forest–swamp and forest–steppe natural zones. We also discuss the effectiveness and practical application of the testate amoebae metabarcoding in the study of ecological problems, including those related to global warming and biosafety.

## MATERIALS AND METHODS

### Sampling locations

**Fig 1:**
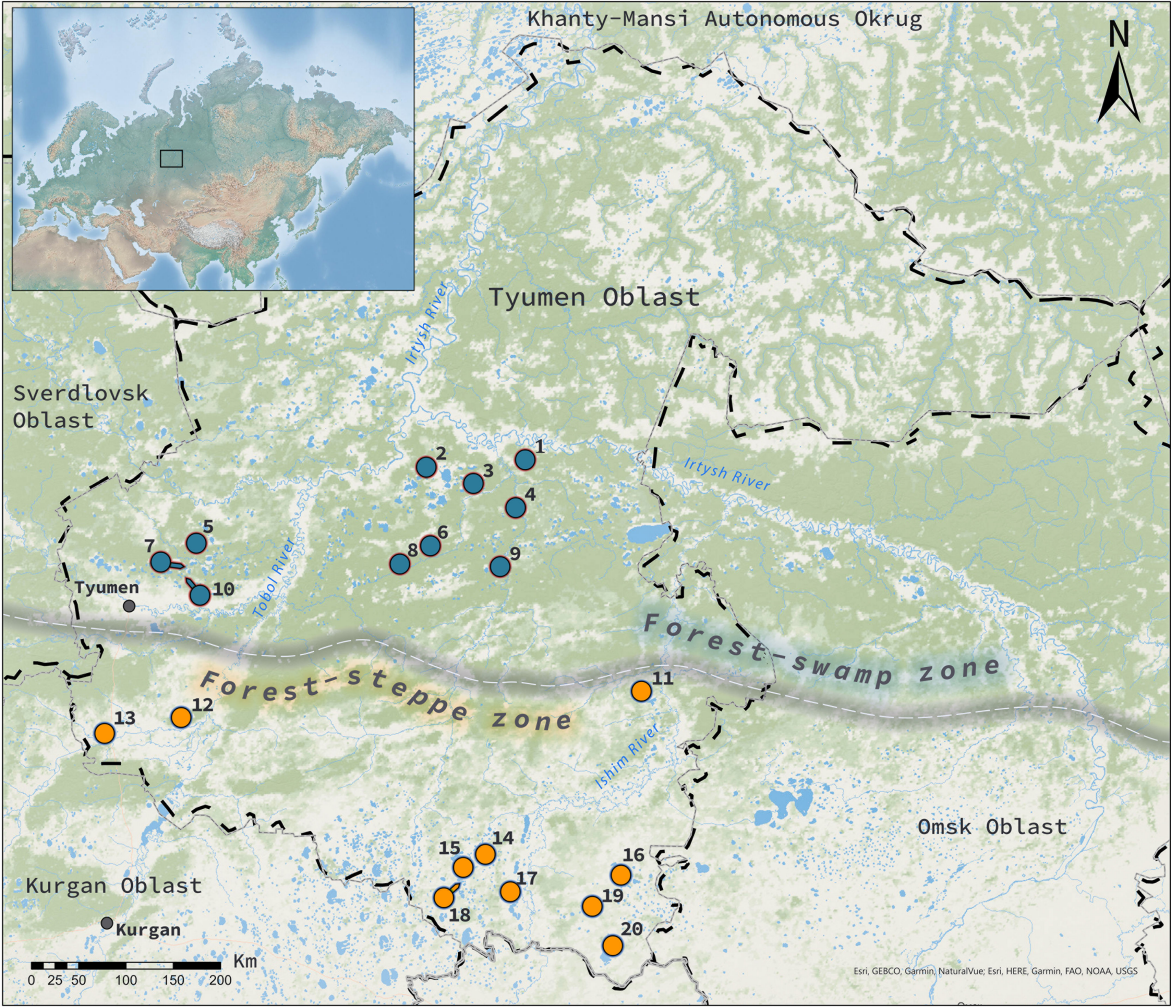
Map of the sampling sites. The numbers correspond to the site numbers in Table 1. The blue points mark the studied lakes in the forest–swamp zone, and the orange points indicate the lakes in the forest–steppe zone. The gray line marks the boundary of the natural zones. The black dashed lines mark the boundaries of the administrative regions. The inset map shows the location of the region of investigations in Russia. The map was sourced from Esri, TomTom, Garmin, FAO, NOAA, USGS; Esri, GEBCO, Garmin, NaturalVue in ArcGIS Pro 3.0.1 software.

The studied lakes are located in Tyumen Oblast of Western Siberia in two natural zones (forest–swamp and forest–steppe) and two landscape provinces (Nizhnetobolsk and Ishim, respectively) that differ in terms of mesoclimatic parameters (Fig. 1; Table 1). Plankton samples from 20 lakes, 10 in each natural zone, were collected on 14–26 June 2022. In each lake, samples were taken in the pelagic zone, in the open littoral zone, and in the littoral zone overgrown with higher aquatic plants. We also collected benthic samples to identify testate amoebae species common to both plankton and benthos and to control the quality of the sampling method.

**TABLE 1:** Characteristics of the studied lakes

No.	Site name	Latitude	Longitude	Salinity (‰)	Total dissolved solids (mg/L)	pH
1	Dikoe	57.914925	69.339705	0.1	145	8.3
2	Svetloe	57.872276	68.368968	0.1	143	8.1
3	Urashnoe	57.791549	68.791258	0.11	147	8.0
4	Maloe	57.649369	69.105488	0.02	35	7.1
5	Ushakovo	57.479233	68.382954	0.1	145	8.0
6	Bolshoe Eleiskoe	57.482686	66.206055	0.08	108	7.8
7	Yurminskoe	57.372768	68.103154	0.19	254	8.1
8	Kuchak	57.351392	66.062272	0.16	216	8.1
9	Chertankul	57.323886	66.036673	0.01	18	6.1
10	Malyi Kuchum	57.268912	69.004440	0.02	26	7.0
11	Kalgan	56.738592	70.346682	0.23	312	8.2
12	Singul	56.587562	66.033969	0.25	334	8.5
13	Terenkul	56.492211	65.314751	0.36	485	8.4
14	Ugryumovo	55.841738	68.918223	0.51	675	8.4
15	Bolshoe Karkovo	55.802614	68.703754	2.43	2,967	8.4
16	Berdyuzhe	55.726463	68.683191	1.43	1,790	8.4
17	Travnoe	55.711713	70.245831	0.33	444	8.2
18	Bezrybnoe	55.602318	69.159687	1.34	1,682	8.3
19	Bolshoi Kurtal	55.577344	69.910598	0.55	719	8.3
20	Solyonoe	55.357978	70.107343	10.97	11,995	8.1

#### Lakes of the forest–swamp zone of the Nizhnetobolsk landscape province

Lakes Dikoe, Svetloe, Urashnoe, Maloe, Bolshoe Eleiskoe, Ushakovo, Yurminskoe, Kuchak, Chertankul, and Malyi Kuchum are located in the forest–swamp zone and have mineralizations ranging from 18 to 254 mg/L and salinities ranging from 0.01‰ to 0.19‰. These lakes are located at the junction of the Southern Taiga subzone and the pine small-leaved forest subzone of the forest–swamp natural zone. The landscape province is located on the left bank of the Irtysh River in the lower reaches of the Tobol and Ishim Rivers. Its territory is dominated by flat plains composed of alluvial sediments. Alluvial sediments are often overlain by loams.

The climate of the Nizhnetobolsk landscape province is moderately cold. The average January temperatures here are not lower than –20°C. In July, the average temperature ranges from 18.0°C–18.5°C. The maximum temperature reached 37°C. Humidification is moderate, and precipitation is 350–430 mm, including 280–350 mm in the warm season. In winter, the thickness of the snow cover does not exceed 50 cm. Most of the area of the Nizhnetobolsk landscape province is occupied by birch forests with a significant distribution of *Sphagnum*, reed, and sedge bogs (30).

#### Lakes of the forest–steppe zone of the Ishim landscape province

Lakes Kalgan, Singul, Terenkul, Ugryumovo, Bolshoe Karkovo, Berdyuzhe, Travnoe, Bezrybnoe, Bolshoi Kurtal, and Solyonoe are located in the forest–steppe zone and have mineralizations ranging from 312 to 11,995 mg/L and salinities ranging from 0.23‰ to 11.0‰.

The Ishim landscape province occupies the territory of the Ishim plain, the surface of which is composed of Neogene sand–clay sediments that are overlain by a thin loam cover.

The climate of the province is characterized by “average” conditions for the forest–steppe zone. The amplitude of average monthly temperatures is 37°C–38°C. Winter is moderately cold; the average January temperature is –18°C to 20°C, and the most severe frosts are –48°C to 52°C. Summer is warm; the average July temperature is 18.5°C–19.5°C; maximum temperatures reach 38°C–40°C. Humidification is not stable; 300–400 mm of precipitation falls throughout the year, mainly during the warm period (250–300 mm). In the second half of winter, the thickness of the snow cover reaches 30–45 cm, but it lies unevenly.

The landscape province is characterized by an abundance of lakes, many of which have salty water. Most of the lakes are chloride, but there are also hydrocarbonate lakes. The most mineralized lakes have a sodium chloride composition (31).

### Sample collection

For DNA analysis, water samples were sequentially filtered through a net with a mesh size of 70 µm and then through nitrocellulose membranes with a pore size of 0.2 µm using a device for vacuum filtering, PVF–35 (Vladisart, Vladimir, Russia). After filtration, each membrane filter was placed in a 1.5 mL Eppendorf tube and stored in 400 µL of 2× DNA/RNA Shield (Zymo Research, California, USA; Cat. No. R1200–125) at +4°C throughout the expedition. At the same time, samples were taken into sterile 50 mL tubes to study the diversity of testate amoebae by light microscopy.

Simultaneously with the collection of biological material, the hydrogen ion concentration index (pH), total dissolved solids, and salinity were measured using a multiparameter sonde YSI EXO2 (YSI Inc., Yellow Springs, OH, USA) (Table 1).

### Light and electron microscopy

An Axio Observer 5 inverted light microscope (Carl Zeiss, Jena, Germany) with phase and differential interference contrasts (20×, 40×, 63× objectives) and an AxioScope A1 upright light microscope (Carl Zeissy) with phase contrast and a water-immersion objective (63×) were used to observe the testate amoebae. Light microscopy images were obtained using MC–12 and MC–20 cameras (Lomo Microsystems, St. Petersburg, Russia). Sample analysis was carried out in Petri dishes with 10–15 mL of the sample. Species identification of testate amoebae was carried out using the monographs Mazei and Tsyganov (32) and Todorov and Bankov (33) and the online database *Microworld, World of Amoeboid Organisms* (34). The morphological characteristics of the studied protists were measured using ImageJ v.1.52a.

### Electron microscopy

The shells of testate amoebae were transferred using a glass micropipette into a drop of distilled water on a coverslip for washing. Small drops containing the shells were transferred to a piece of polycarbonate membrane with a pore size of 0.8 µm glued with a conductive adhesive tape on an aluminium SEM specimen stub. After air drying, the preparation was coated with a layer of gold using Auto Fine Coater JFC–1600 (JEOL, Tokyo, Japan) and observed with a JSM–6510LV (JEOLan) scanning electron microscope at an acceleration voltage of 15–30 kV.

### eDNA extraction, amplification, and 18S rRNA gene library preparation

The samples were stored in the laboratory at –20°C until DNA extraction. DNA was extracted using the Quick–DNA Fecal/Soil Microbe MiniPrep Kit (Cat. No. D6010) according to the manufacturer’s instructions, but with double DNA washing. For DNA extraction, each membrane and 100 µL of 2× DNA/RNA Shield were transferred to a ZR Bashing Bead (0.1 and 0.5 mm) Lysis Tube (Zymo Research, Cat. No. D6010) with the addition of 750 µL of BashingBead Buffer and homogenized for 15 min at 50 Hz (TissueLyser LT; Qiagen, Germany).

The quality of the extracted DNA was checked using electrophoresis in a 1% agarose gel. The concentration of DNA was quantified with a Qubit 4 fluorometer (Invitrogen, USA) using a Qubit 1× dsDNA HS Assay Kit (Invitrogen). Nuclease-free water (Qiagen, Cat. No. 129115) was used as a negative control for water sample filtration.

Fragments encompassing the V4 region of the 18S rRNA gene were amplified using the universal non-metazoan primers:

18S_EUK–565F 5′-GCAGTTAAAAAGCTCGTAGT-3′ (35);
18S_EUK–1134R 5′-TTTAAGTTTCAGCCTTGCG-3′ (36).

Further sequencing was performed using the Illumina MiSeq platform and MiSeq Reagent Kit v.3 2 × 300 bp (Illumina) according to the Illumina workflow (Illumina protocol, Part No. 15044223, Rev. B).

### eDNA analysis

The quality of the sequenced reads was checked using FastQC v.0.11.9 (37). Cutadapt v.3.5 (38) was used for primer sequence removal from the reads. The DADA2 pipeline (39) was used for further sequence analysis, including quality filtering, read merging (minimum overlap = 18 bp), chimera removal, and amplicon sequence variant (ASV) generation. Reads were truncated to lengths of 290 and 250 bp from R1 and R2 reads, respectively, and filtered with maximum expected errors of 2 and 4 for forward and reverse reads, respectively. Taxonomy was assigned to each ASV using the PR2 database v.4.14.0 (40). After taxonomic assignment, all unclassified to domain and phylum levels ASVs were manually classified by BLASTn with standard parameters. ASVs with singletons were removed to minimize the impact of likely spurious sequences. Only ASVs classified as testate amoebae were analyzed.

### Phylogenetic analysis of testate amoeba ASVs

Sequences of ASVs of testate amoebae and their related 18S rRNA gene sequences from National Center for Biotechnology Information (NCBI) were aligned using the L–INS–i algorithm in MAFFT v.7.475 (41) and trimmed using the “–gappyout” method in TrimAl v.1.2 (42). All phylogenetic analyses were performed on two data sets.

#### Emphasis on Imbricatea

Maximum-likelihood phylogeny for 123 sequences (including 9 ASVs and 5 Endomyxa as the outgroup) was inferred using IQ–TREE v.1.6.12 with 1,816 unambiguously aligned positions and 1,000 non-parametric bootstraps under the best fit model (TN + F + I + G4) determined by the in-built ModelFinder. To infer the Bayesian phylogenetic tree (43), MrBayes v.3.2.7 a (13) was used with four categories of gamma-distributed among-site rate variation under the GTR + I + GAMMA4 substitution model, calculating the proportion of invariable sites. To calculate posterior probability, four independent Metropolis-coupled Markov chains were run for 20 million generations and summarized with a 50% burn-in. Convergence of log-likelihood values and model parameters for chains was verified using a plot and convergence diagnostics provided by the MrBayes “sump” utility. The average standard deviation of the bipartition frequencies was recorded at 0.016 by the end of the run.

#### Emphasis on Thecofilosea

Maximum-likelihood phylogeny for 121 sequences (including 11 ASVs and 5 Endomyxa as the outgroup) was inferred using IQ–TREE v.1.6.12 with 1,695 unambiguously aligned positions and 1,000 non-parametric bootstraps under the best fit model (TIM3 + F + R4) determined by the in-built ModelFinder. Bayesian analysis was performed with the same parameters as for the first data set. The average standard deviation of the bipartition frequencies was recorded at 0.017 by the end of the run.

### Data analysis

Analysis and visualization of morphospecies and ASVs data were performed using the R statistical programming language (R Core Team, 2021) in the RStudio environment. To construct the rarefaction curve and perform hierarchical clustering, functions from the vegan package were used (44). The distance matrix based on the Bray–Curtis distances was calculated using the *vegdist* function. To study the correlation between hydrological variables and the community structure of testate amoebae, canonical correlation analysis (CCA) was performed using the *cca* function. To assess the influence of the factor under study on the dependent variable, analysis of variance was performed using the *anova* function. The calculation results were considered statistically significant at *P* < 0.05. The *draw.pairwise.venn* function from the VennDiagram package (45) was used to construct a Venn diagram.

## RESULTS

### Diversity of planktonic testate amoebae according to microscopy

Forty species of planktonic testate amoebae were revealed in lakes of the forest–swamp and forest–steppe natural zones using light microscopy (Table 2). Of these, 10 species (*Alabasta militaris*, *Archerella flavum*, *Arcella hemisphaerica*, *Cryptodifflugia sacculus*, *C. vulgaris*, *Frenzelina reniformis*, *Heleopera rosea*, *Hyalosphenia papilio*, *Phryganella dissimulatoris*, and *Physochila griseola*) were identified only in the forest–swamp zone lakes, and 17 species were identified only in the forest–steppe zone lakes (*Arcella hemisphaerica playfairiana*, *Campascus interstitialis*, *Centropyxis aculeata*, *C. aculeata minima*, *C. aerophila*, *Cryptodifflugia crenulata*, *C. sacculus sakotschawi*, *C. minuta*, *C. voigti*, *Cylindrifflugia elegans*, *Cyphoderia ampulla*, *C. laevis*, *Difflugia louisi*, *Euglypha tuberculata*, *Meisterfeldia wegeneri*, *Pseudodifflugia archeri*, and *Trinema lineare*).

**TABLE 2:** Species composition and frequency of occurrence (%) of planktonic testate amoebae of lakes in the forest–swamp and forest–steppe zones in Western Siberia^*a*^^,^^*b*^

	Lakes of the forest–swamp zone										Lakes of the forest–steppe zone									
	Dikoe	Svetloe	Urashnoe	Maloe	Ushakovo	Bolshoe Eleiskoe	Yurminskoe	Kuchak	Chertankul	Malyi Kuchum	Kalgan	Singul	Terenkul	Ugryumovo	Bolshoe Karkovo	Berdyuzhe	Travnoe	Bezrybnoe	Bolshoi Kurtal	Solyonoe
Amorphea Adl et al., 2012																				
*Amoebozoa Lühe, 1913, sensu Cavalier-Smith, 1998																				
**Tubulinea Smirnov et al., 2005																				
***Elardia Kang et al., 2017																				
****Arcellinida Kent, 1880																				
*****Glutinoconcha Lahr et al., 2019, suborder																				
******Sphaerothecina Kosakyan et al., 2016, infraorder																				
*******Arcellidae Ehrenberg, 1843, family																				
********Arcella Ehrenberg, 1832, genus																				
*********Arcella hemisphaerica **Perty, 1852**				33					33											
*********Arcella hemisphaerica playfairiana **Deflandre, 1928**																33				
*********Arcella hemisphaerica undulata **Deflandre, 1928**				33					33		33									
*****Organoconcha Lahr et al., 2019, suborder																				
******Microchlamyiidae Ogden 1985, family																				
*******Microchlamys Cockerell 1911, genus																				
********Microchlamys patella **(Claparède and Lachmann, 1859) Cockerell, 1911**	67			67		33			33		33		33		33		33		33	
*******Pyxidicula Ehrenberg, 1838, genus																				
********Pyxidicula operculata **(Agardh, 1827) Ehrenberg, 1838**	67		67	33	33	67		33			33			33			67			
******Longithecina Lahr et al., 2019, infraorder																				
*******Difflugiidae Wallich, 1864, family																				
********Difflugia Leclerc, 1815, genus																				
*********Difflugia louisi **(Chardez and Beyens, 1988)**											33									
******Excentrostoma Lahr et al., 2019, infraorder																				
*******Centropyxidae Jung, 1942, family																				
********Centropyxis Stein, 1857, genus																				
*********Centropyxis aculeata **(Ehrenberg, 1838) Stein, 1859**																	33			
*********Centropyxis aculeata minima **van Oye, 1958**																	33			
*********Centropyxis aerophila **Deflandre, 1929**											33									
******Volnustoma Lahr et al., 2019, infraorder																				
*******Heleoperidae Jung, 1942, family																				
********Heleopera Leidy, 1879, genus																				
*********Heleopera rosea **Penard, 1890**									33											
******Hyalospheniformes Lahr et al., 2019, infraorder																				
*******Hyalospheniidae Schultze, 1877 emend (Kosakyan et al., 2012), family																				
********Hyalosphenia Stein, 1859, genus																				
*********Hyalosphenia papilio **Leidy, 1874**									33											
********Alabasta Duckert et al., 2018, genus																				
*********Alabasta militaris **(Penard, 1890) Duckert et al., 2018**									33											
******Cylindrothecina González-Miguéns et al., 2022, infraorder																				
*******Cylindrifflugiidae González-Miguéns et al., 2022, family																				
********Cylindrifflugia González-Miguéns et al., 2022, genus																				
*********Cylindrifflugia elegans **(Penard, 1890) González-Miguéns et al., 2022**																	33			
*****Phryganellina Bovee, 1985, suborder																				
******Phryganellidae Jung, 1942, family																				
*******Phryganella Penard, 1902, genus																				
********Phryganella acropodia **(Hertwig and Lesser, 1874) Hopkinson, 1909**		33				33			67		33		33							
********Phryganella dissimulatoris **Chardez, 1969**					33															
******Cryptodifflugiidae Jung, 1942, family																				
*******Cryptodifflugia Penard, 1890, genus																				
********Cryptodifflugia compressa **Penard, 1902**			33				33			33		33								
********Cryptodifflugia crenulata **Playfair, 1917**											33					33	33			
********Cryptodifflugia sacculus **Penard, 1902**			33		33				33											
********Cryptodifflugia sacculus sakotschawi **Tarnogradsky, 1959**																		33		
********Cryptodifflugia horrida **Page, 1966**			33														33	67		
********Cryptodifflugia minuta **Playfair, 1917**																				33
********Cryptodifflugia oviformis **Penard, 1902**						33			33			33								
********Cryptodifflugia voigti **(Golemansky, 1970) Bobrov et al., 2017**																33				
********Cryptodifflugia vulgaris **(Francé, 1913) Volz, 1928**				33																
********Cryptodifflugia patinata **(Schönborn, 1965) Bobrov et al., 2017**					100			33											33	
********Cryptodifflugia pusilla **Playfair, 1917**			67		33			33			33					33			33	
Arcellinida incertae sedis																				
******Physochila Jung, 1942, genus																				
*******Physochila griseola **(Wailes and Penard, 1911)**									33											
******Meisterfeldia Bobrov, 2016, genus																				
*******Meisterfeldia wegeneri **Bobrov, 2016**													33			33				
Diaphoretickes Adl et al., 2012																				
Sar Burki et al., 2008, emend. Adl et al., 2012																				
*Rhizaria Cavalier-Smith, 2002																				
**Cercozoa Cavalier-Smith, 1998, emend. Adl et al., 2005; emend. Cavalier-Smith, 2018																				
***Imbricatea Cavalier- Smith, 2011 (Cavalier-Smith, 2003)																				
****Silicofilosea Adl et al., 2005, emend. Adl et al., 2012																				
*****Euglyphida Copeland 1956, emend. Cavalier-Smith, 1997 Order																				
******Cyphoderiidae de Saedeleer, 1934, family																				
*******Campascus Leidy, 1879, genus																				
********Campascus interstitialis **Golemansky, 1981**																67				67
*******Cyphoderia Schlumberger, 1845, genus																				
********Cyphoderia ampulla **(Ehrenberg, 1840)**															33					
********Cyphoderia laevis **Penard, 1902**																	33			
******Euglyphina Kosakyan et al., 2016, suborder																				
*******Euglyphidae Wallich, 1864, emend Lara et al., 2007, family																				
********Euglypha Dujardin, 1841, genus																				
*********Euglypha tuberculata **Dujardin, 1841**																	33			
*******Trinematidae Hoogenraad and De Groot, 1940, family																				
********Trinema Dujardin, 1841, genus																				
*********Trinema lineare **Penard, 1890**																	33			
***Thecofilosea Cavalier-Smith 2003, emend. Cavalier-Smith, 2011																				
****Tectofilosida Cavalier-Smith, 2003																				
*****Chlamydophryidae de Saedeleer, 1934, family																				
******Lecythium Hertwig and Lesser, 1876, genus																				
*******Lecythium hyalinum **Hertwig and Lesser, 1874 emend. Dumack et al., 2016**		33		33								67		33		33			33	
Incertae sedis cercozoan testate amoebae																				
***Frenzelina Penard, 1902, genus																				
****Frenzelina minima **Hoogenraad, 1910**			33	33		67	33	33	67			67								
****Frenzelina reniformis **Penard, 1902**							33													
***Pseudodifflugiidae De Sandeleer, 1934, family																				
****Pseudodifflugia Schlumberger, 1845, genus																				
*****Pseudodifflugia archeri **Penard, 1899**																	33			
*****Pseudodifflugia klarae **Kiss and Török, 2009**					67		33		33	33	67		67	100	33	67	100	33	33	33
*****Pseudodifflugia simensmai **sp. nov.**						67					33	67	33						33	
*Stramenopiles Patterson 1989, emend. Adl et al., 2005																				
**Bigyra Cavalier-Smith 1998, emend., 2006																				
***Sagenista Cavalier-Smith, 1995																				
****Labyrinthulomycetes Dick, 2001																				
*****Amphitremida Poche, 1913, emend. Gomaa et al., 2003																				
******Archerella Loeblich and Tappan, 1961, genus																				
*******Archerella flavum **(Archer, 1877) Loeblich and Tappan, 1961**									33											

^*a*^ Asterisks indicate taxonomic categories, with a greater number of asterisks corresponding to lower taxonomic ranks.

^*b*^ Species names are indicated in bold.

Testate amoebae *Campascus interstitialis*, *Cryptodifflugia sacculus sakotschawi*, *C. voigti*, and *Cyphoderia ampulla* were detected only in lakes of the forest–steppe zone with salinities of 1‰–11‰ (lakes Bolshoe Karkovo, Berdyuzhe, Bezrybnoe, and Solyonoe).

**Fig 2:**
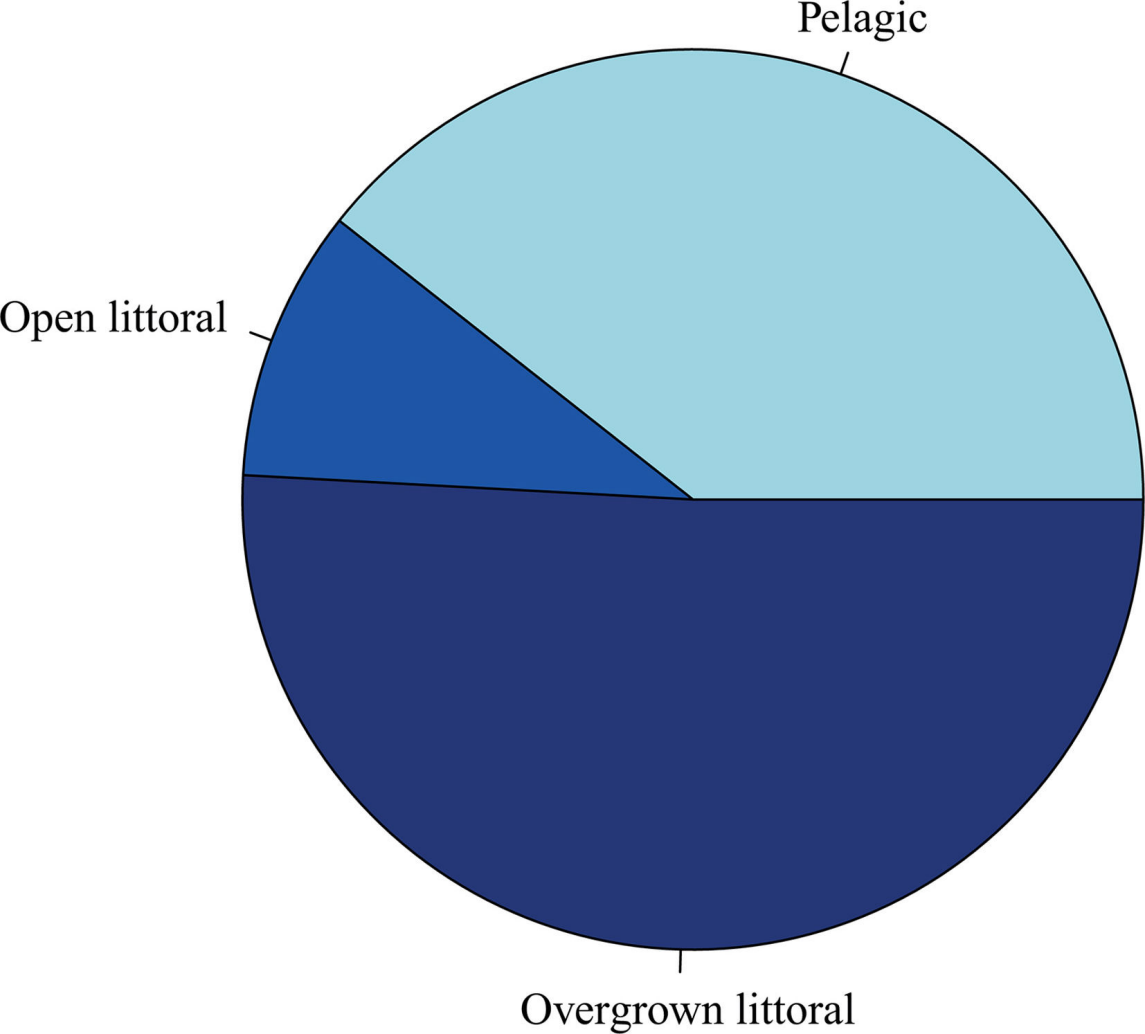
Percentages of ASVs in the sampling zones within the waterbodies.

Considering the different zones within the lakes, the number of planktonic species of testate amoebae increased in the following order: open littoral, pelagial, and overgrown littoral. The number of species in the overgrown littoral zone was more than 2.6 times higher than that in the pelagic zone and 2.7 times higher than that in the open littoral zone (Fig. 2).

### A new species of the genus *Pseudodifflugia*

A new species of testate amoeba was revealed in the plankton of freshwater lakes Bolshoe Eleiskoe, Svetloe, Dikoe, Bolshoi Kurtal, Kalgan, Singul, and Terenkul.

Rhizaria Cavalier-Smith, 2002

Cercozoa Cavalier-Smith, 1998, emend. Adl et al., 2005; emend. Cavalier-Smith, 2018

Cercozoa *incertae sedis*

Fam. Pseudodifflugiidae De Saedeleer, 1934

Gen. *Pseudodifflugia* Schlumberger, 1845

#### Pseudodifflugia siemensmai Zagumyonnaya, Zagumyonnyi et Tikhonenkov sp. nov.

##### Description

**Fig 3:**
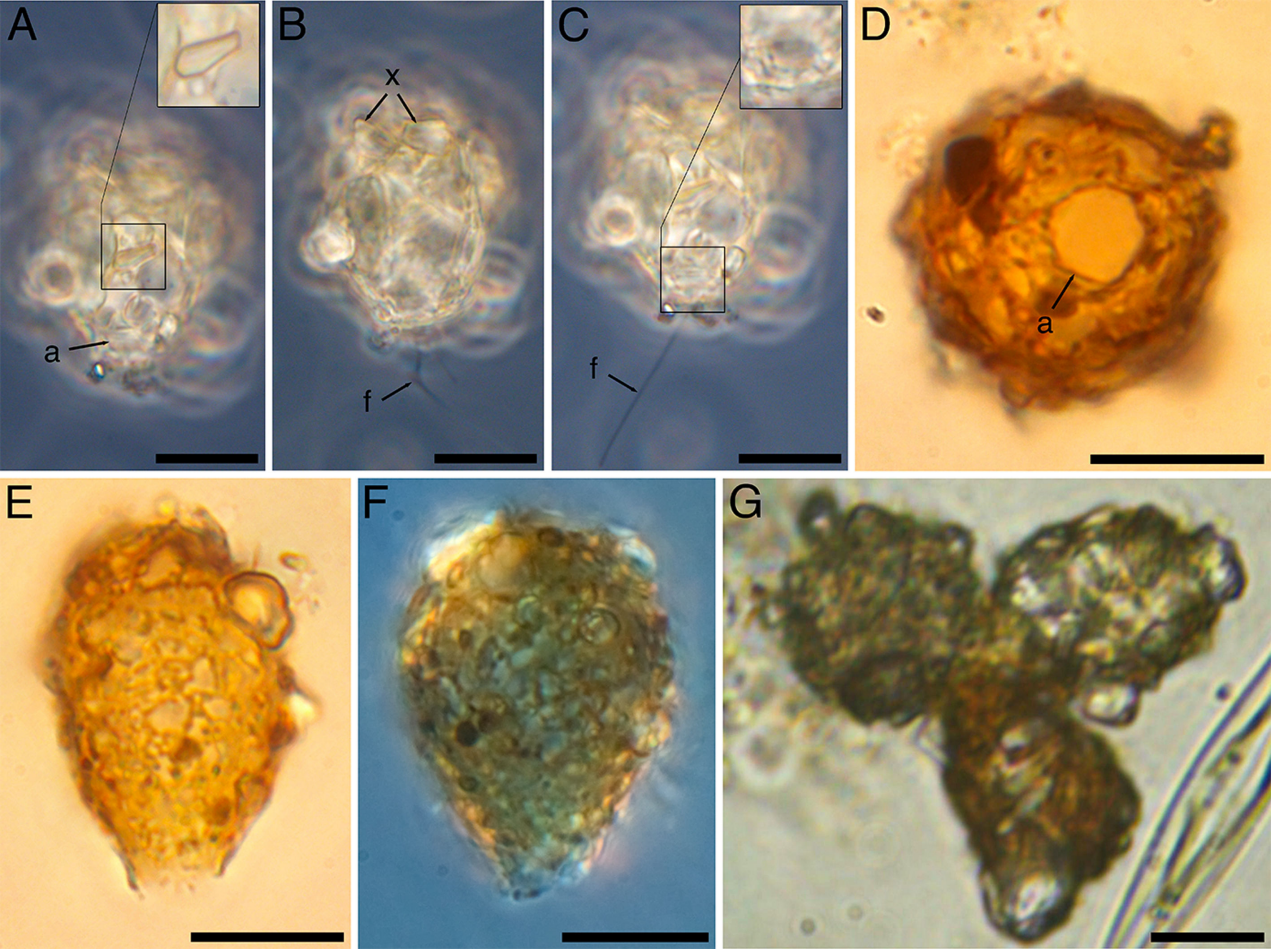
*Pseudodifflugia siemensmai* sp. nov. (A–C) in phase contrast and (D–G) in bright field microscopy. Images D–F are stacked from multiple images. (A–C) General view of the living cell. The inset in panel A shows the xenosome. The inset in panel С shows the aperture. (**D**) View of the apical part of the shell with the aperture. (E and F) General view of the shells. (**G**) Group of shells in “'rosett” formation. Scale bars: 10 µm. Abbreviations: a, aperture; f, filopodia; x, xenosome.

The cells with a pyriform, uncompressed shell (Fig. 3). The shell sizes varied from 18.4 to 26.8 (average of 23.9) ×14.2–18.9 (average of 17.8) µm. The length-to-width ratio is 1.3–1.5. The shell is agglutinated with transparent or dark-brown mineral 0.3–10.9 µm-long xenosomes (Fig. 3B). The aperture is circular, without a collar, with a diameter of 4.7–5.9 (average of 5.4) µm (Fig. 3A). The filopodia branch but do not anastomose. The length of the filopodia ranged from three to four times the size of the shell. Shells can be aggregated into “rosette” formations (Fig. 3G).

##### Diagnosis

Filopodia branch but do not anastomose. The shell is 18.4–26.8 × 14.2–18.9 µm, pyriform, not compressed, and consists of xenosomes of various shapes and sizes. The length-to-width ratio is 1.3–1.5. The aperture is circular, without a collar, with a diameter of 4.7–5.9 µm.

##### Differences from other species

The morphological characteristics of this species are most similar to those of the testate amoeba *Pseudodifflugia* spec. 1 from the sediments of the peat bog Bert Bospad (Netherlands) published in the online database (Siemensma, F. J., Microworld, world of amoeboid organisms, World-wide Electronic Publication, Kortenhoef, the Netherlands; searched on 11 March 2024). The studied organism is distinguished by its larger size (18.4–26.8 × 14.2–18.9 µm vs. 15.7–12.0 µm) and a larger aperture (4.7–5.9 µm vs. 3.4 µm [measured from photo]). The shell shape is similar to that of *Pseudodifflugia microstoma* Playfair, 1917, but differs in its smaller size (18.4–26.8 × 14.2–18.9 vs. 30–31 × 17–23 µm) (46). Snegovaya and Alekperov (47) proposed a new combination where *P. microstoma* was transferred to the genus *Lenkorania* Snegovaya et Alekperov, 2010. The organism from the water reservoirs of the Lankaran Natural Area (Azerbaijan) studied by the authors also had a larger shell size (55–60 × 40–45 µm) and aperture size (8–9 µm) but smaller xenosomes relative to the shell size. In contrast, another similar species, *Pseudodifflugia klarae* Kiss et Török, 2009, is characterized by its smaller size (8–14 µm) and irregular aperture, 0.4–3.0 µm in diameter (48). *Pseudodifflugia fulva* (Archer 1870) (49), which is close to the species we studied in shell size (15–23 µm), is characterized by a wider aperture (7.5–11.5 µm) (49).

##### Etymology

The species name is dedicated to the researcher Ferry Siemensma in recognition of his contributions to the study of testate amebae.

##### Hapantotype

Preparation for SEM No. T1–Ps.1 is kept in the Laboratory of Microbiology at Papanin Institute for Biology of Inland Waters, Russian Academy of Sciences (Borok, Russia).

##### Type figure

See Fig. 4.

**Fig 4:**
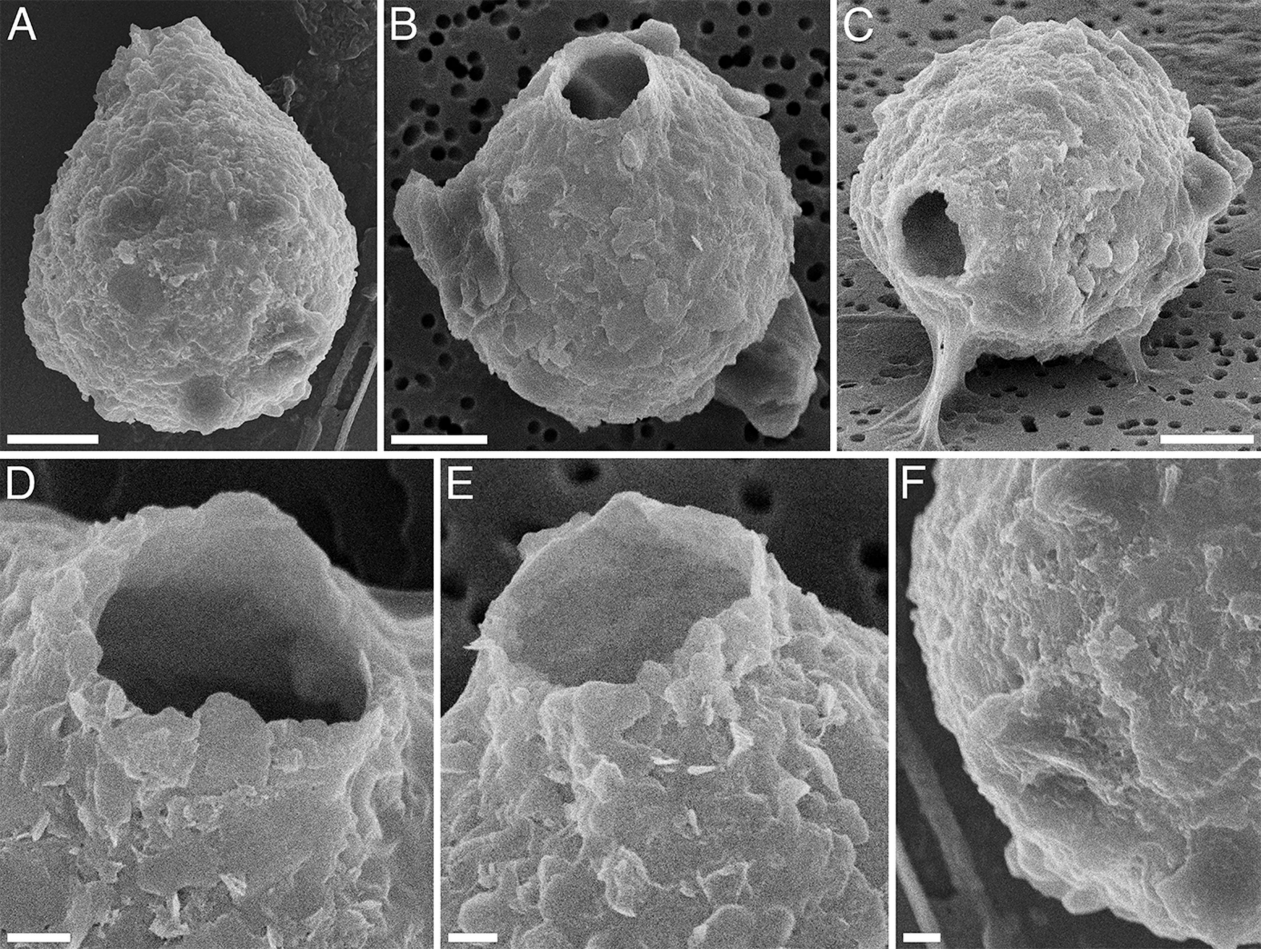
Scanning electron microscopy of *Pseudodifflugia siemensmai* sp. nov. (A–C) General view of shells. (D and E) Aperture. (**F**) Distal part of the shell. Scale bars: 1 µm (D–F) and 5 µm (A–C).

##### Type locality

The species belongs to the plankton of the overgrown littoral zone of Lake Singul, Tyumen region, Western Siberia, 56°57′76 N, 66°04′60 E.

##### Zoobank Life Science Identifier (LSID) of the species

The Zoobank LSID of the species is urn:lsid:zoobank.org:act:8619CC01-9CE7-44E2-BC85-B240A79137D2.

##### Zoobank LSID of the publication

The Zoobank LSID of the publication is urn:lsid:zoobank.org:pub:CB0CFA54-8DBE-4271–8445-19CF233B70BD.

### Diversity of testate amoebae according to V4 18S rRNA metabarcoding

**Fig 5:**
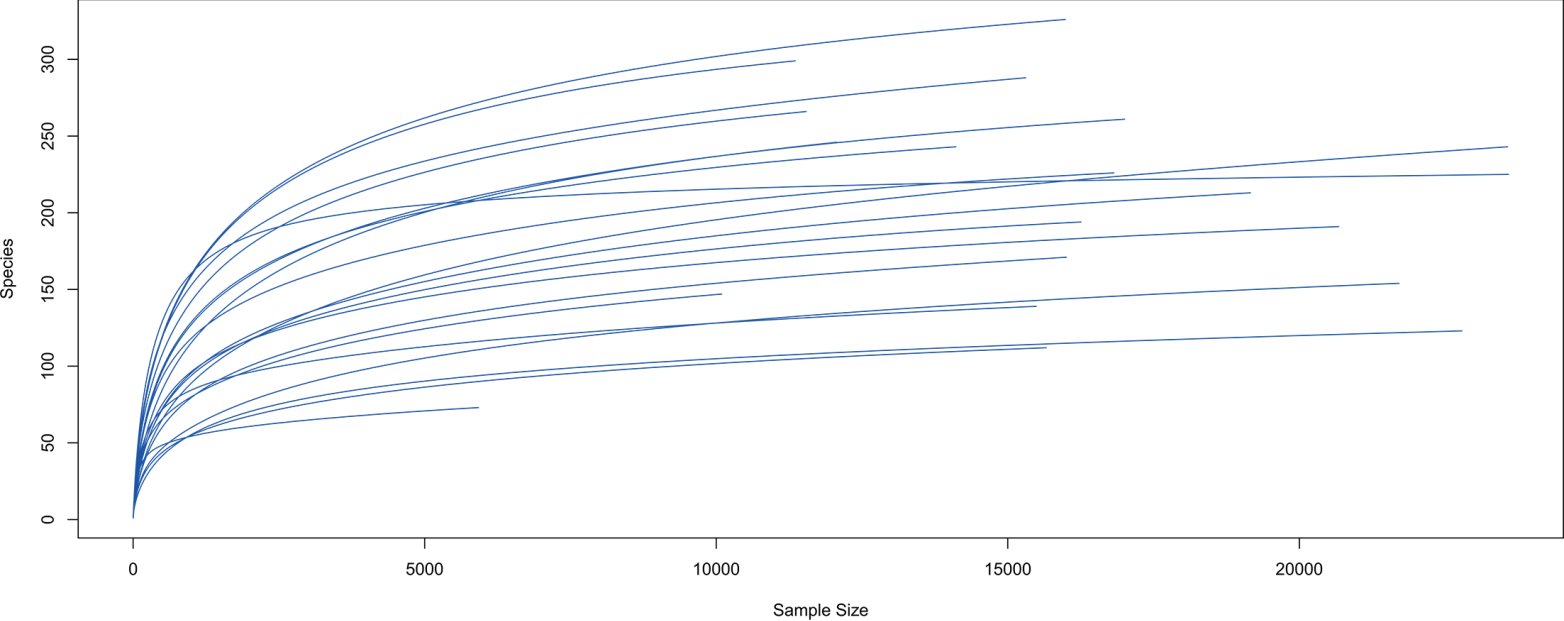
Rarefaction curve of sequenced libraries plotted between the total number of reads (*x*-axis) and the number of amplicon sequence variants (*y*-axis).

A total of 8,757 protist ASVs were obtained using the DADA2 pipeline after removing non-specific amplification sequences, quality filtering, dereplication, chimera removal, and read merging. Rarefaction curves of each sample gradually reached a plateau after 5,000 sequences, indicating that the data set was sufficient for the diversity analysis (Fig. 5).

High–throughput sequencing and bioinformatics processing, including additional BLASTn search, revealed 20 testate amoeba ASVs belonging to Cercozoa (Rhizaria), while no testate amoeba ASVs were found within either Stramenopiles or Amoebozoa.

Of the 20 lakes studied, ASVs of planktonic testate amoebae were found in only 15. The total abundance of 18S rRNA reads of testate amoebae was 348. The largest number of ASV sequences (208) was noted for lakes in the forest–steppe zone compared to lakes in the forest–swamp zone (140 sequences) (Table 3).

**TABLE 3:** Number of ASV reads for testate amoeba communities in forest–swamp and forest–steppe lakes^*a*^

ASV	Forest–swamp lakes						Forest–steppe lakes								
	Dikoe	Malyi Kuchum	Svetloe	Urashnoe	Ushakovo	Chertankul	Kalgan	Bezrybnoe	Berdyuzhe	Bolshoi Kurtal	Singul	Solyonoe	Terenkul	Travnoe	Ugryumovo
1897	-	-	-	-	-	-	-	-	-	-	8	-	-	1	-
16,35	-	-	-	8	1	-	-	1	-	-	-	-	1	2	-
2082	-	-	-	-	-	1	1	3	1	-	-	-	2	-	-
2112	-	-	-	-	-	-	-	-	-	-	-	8	-	-	-
1251	-	-	5	-	-	-	-	-	-	5	11	-	-	-	1
1323	-	-	2	-	-	-	5	-	-	4	-	-	-	-	-
3013	-	-	-	-	-	-	1	2	-	-	-	-	-	-	-
472	-	-	-	-	-	86	-	-	-	-	-	-	-	-	-
1947	-	-	-	9	-	-	-	-	-	-	-	-	-	-	-
1385	-	-	-	-	-	-	-	-	-	-	19	-	-	-	-
1590	-	5	-	-	-	-	-	-	-	-	-	-	-	9	-
2111	-	-	-	-	-	-	-	-	-	-	-	8	-	-	-
2969	-	-	-	-	-	-	-	2	-	1	-	-	-	-	-
1780	-	-	-	2	-	-	6	-	-	-	3	-	-	-	-
2237	-	-	-	-	8	-	-	-	-	-	-	-	-	-	-
2623	-	-	-	-	-	-	-	-	-	2	-	-	-	-	2
886	2	4	-	1	-	-	-	24	6	1	-	-	-	-	1
1034	-	-	-	-	-	-	-	-	-	-	31	-	-	-	-
2284	6	-	-	-	-	-	-	-	-	-	-	-	-	-	-
939	-	-	-	-	-	-	-	-	-	-	-	36	-	-	-
Total number of ASV reads	140						208								

^*a*^ – indicates absence of ASV.

The number of sequences of ASVs of planktonic testate amoebae was 1.5 times higher for forest–steppe lakes than for forest–swamp zone lakes. The number of revealed testate amoeba ASVs increased in the following order: pelagial, overgrown littoral, and open littoral.

The potentially pathogenic genera of testate amoebae *Rhogostoma* and *Fisculla* were revealed only by the metabarcoding approach. Representatives of the genus *Rhogostoma* were found in 12 of the 20 studied lakes: Bezrybnoye, Berdyuzhe, Bolshoi Kurtal, Kalgan, Svetloye, Singul, Terenkul, Travnoye, Ugryumovo, Urashnoye, Ushakovo, and Solyonoye. Representatives of the genus *Fisculla* were found in two lakes (Kalgan and Bezrybnoye).

### Phylogenetic position of the revealed ASVs

**Fig 6:**
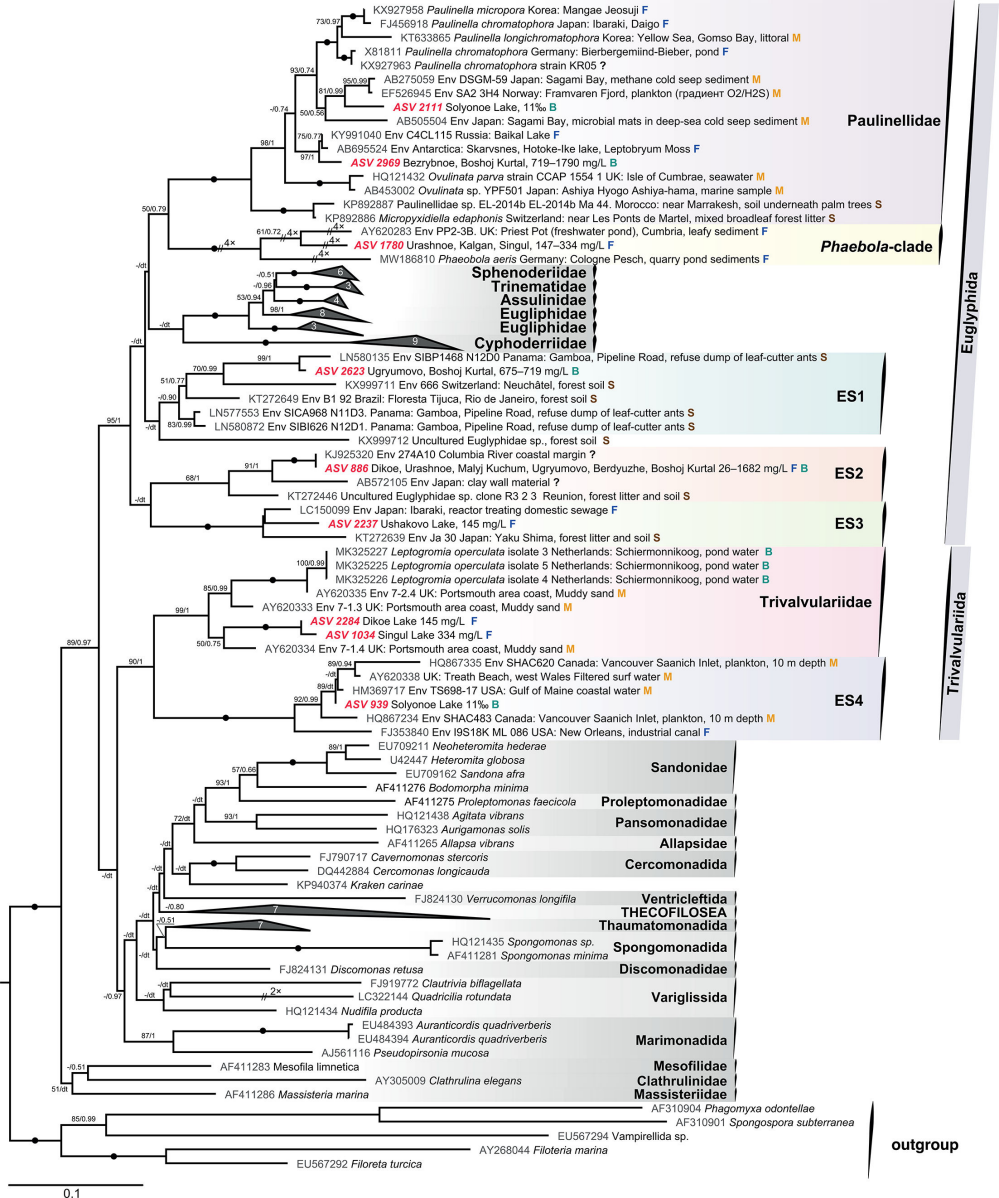
Maximum-likelihood phylogenetic tree of cercozoans focused on Imbricatea 18S rRNA gene sequences. Maximum-likelihood (ML) bootstrap values and Bayesian posterior probabilities (BPPs) are indicated at branches (values greater than 50% are shown); black-filled circles indicate values of BPP = 1.00 and ML bootstrap = 100%. The symbols “//” and “4×” indicate that the branch is shortened four times to improve visualization. The numbers on the collapsed clades indicate the number of sequences included. The ASVs obtained in this study are highlighted in bold and red. At the end of the sequence names, different colored letters indicate the habitat types from which the samples were taken: F (blue), freshwater; S (brown), soil; M (orange), marine water; and B (green), brackish water. dt, different topology.

**Fig 7:**
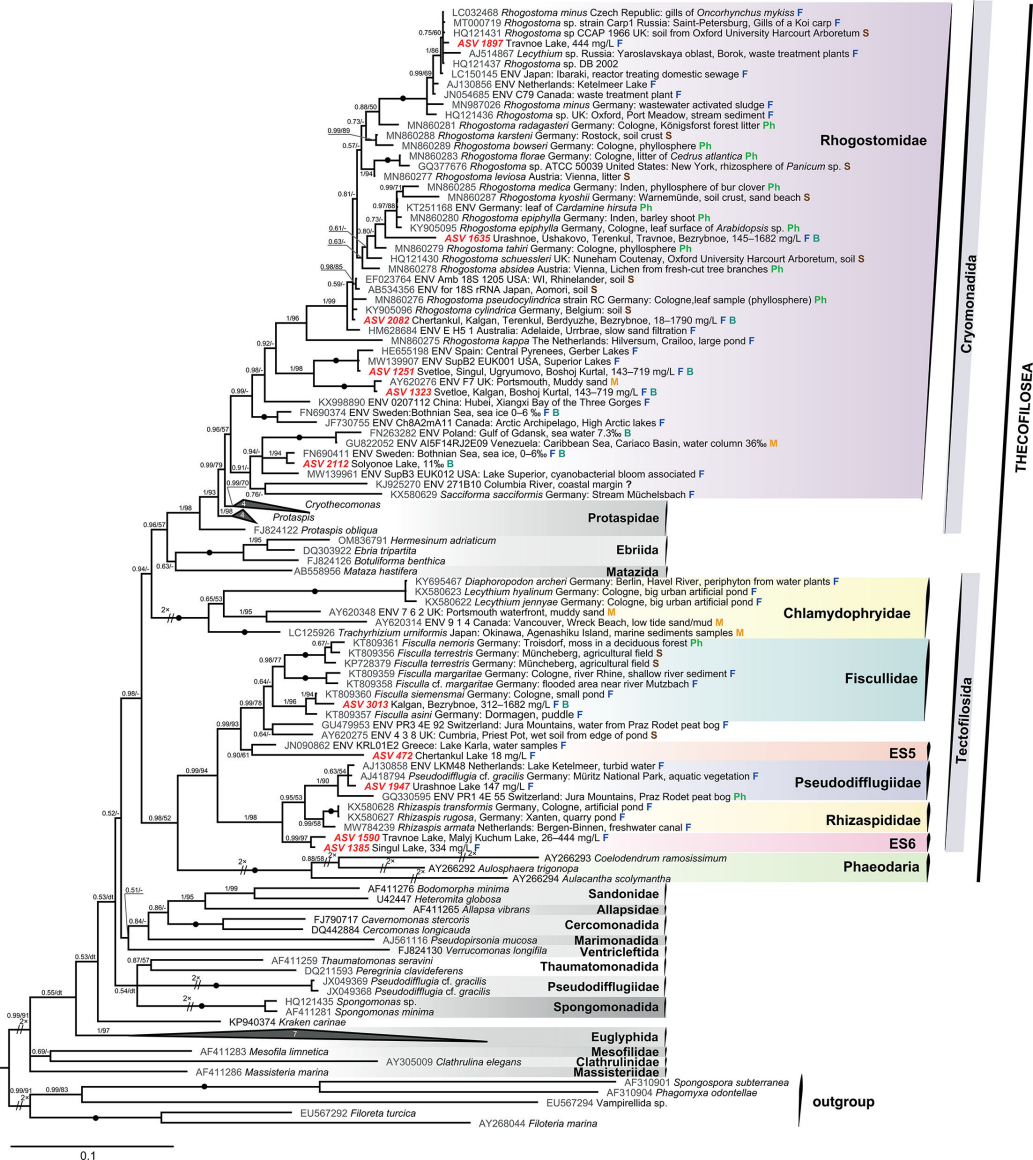
Bayesian phylogenetic tree of cercozoans focused on Thecofilosea 18S rRNA gene sequences. Bayesian posterior probabilities (BPPs) and maximum-likelihood (ML) bootstrap values are indicated at branches (values greater than 50% are shown); black-filled circles indicate values of BPP = 1.00 and ML bootstrap = 100%. The symbols “//” and “2×” indicate that the branch is shortened two times to improve visualization. The numbers on the collapsed clades indicate the number of sequences included. The ASVs obtained in this study are highlighted in bold and red. At the end of the sequence names, different colored letters indicate the habitat types from which the samples were taken: F (blue), freshwater; S (brown), soil; M (orange), marine water; B (dark green), brackish water; and Ph (light green), phyllosphere. dt, different topology.

Only 2 out of 20 revealed ASVs had a match with a known species, namely, ASV 2082 with *Rhogostoma cylindrica* Flues et Dumack, 2017 (KY905096, 99.8% similarity), and, possibly, ASV 1947 with *Pseudodifflugia* cf. *gracilis* Wylezich, Meisterfeld, Meisterfeld and Schlegel, 2002 (AJ418794, 99.4% similarity). Other ASVs did not match the annotated species. Phylogenetic analyses revealed the phylogenetic affiliations of these ASVs of planktonic testate amoebae among Cercozoa. They belonged to the orders Euglyphida and Trivalvulariida within Imbricatea (Fig. 6), and Tectofilosida and Cryomonadida within Thecofilosea (Fig. 7).

Figure 6 shows the grouping of nine ASVs among the order Euglyphida within Paulinellidae, *Phaeobola* clade, environmental sequence clades ES1, ES2, and ES3, and among the order Trivalvularida, within Trivalvulariidae and environmental sequence clade ES4 (Fig. 6). As revealed in this study, clades ES1, ES2, ES3, and ES4 represent the hidden and not annotated phylogenetic diversity of testate amoebae within Cercozoa.

### Order Euglyphida

#### Paulinellidae

This family includes three genera, *Paulinella* Lauterborn, 1895, which has silica idiosomes, and *Ovulinata* Anderson, Rogerson, et Hannah, 1997, and *Micropyxidiella* Tarnawski et Lara, 2015, which have organic shells without idiosomes. ASV 2111 and ASV 2969 are grouped with members of the genus *Paulinella*. ASV 2111 from Solyonoe Lake (11‰) groups with high support (81.0/0.99) in a clade containing marine environmental sequences AB275059 and EF526945 from Japan and Norway. Together with the environmental sequence from the deep-sea microbial mats, this clade is sister to a clade that includes sequences of *Paulinella micropora* Lhee, Yang, Kim, Andersen, et Yoon, 2017, *P. chromatophora* Lauterborn, 1895, and *Paulinella longichromatophora* Kim et Park, 2016. These species are known for possessing plastids (chromatophores) of cyanobacterial origin unique to eukaryotes other than Archaeplastida. ASV 2969 from Bezrybnoe Lake and Bolshoi Kurtal Lake is grouped in a more basal freshwater clade together with the environmental sequences KY991040 and AB695524 from Lake Baikal and Hotoke–Ike Lake in Antarctica.

#### Phaeobola clade

ASV 1780 from Urashnoe Lake, Kalgan Lake, and Singul Lake (147–334 mg/L) was grouped with full support with *Phaeobola aeris* Dumack et al., 2020, and an environmental sequence from the freshwater pond Priest Pot, UK (AY620283). *P. aeris* is the first morphologically identified representative of previously revealed cryptic diversity (“dark matter”) of Euglyphida (14). This species lacks the silica idiosomes typical of Euglyphida members (50).

##### ES1

ASV 2623 from Ugryumovo Lake and Bolshoi Kurtal Lake (675–719 mg/L) clusters with high support (99/1) with the environmental sequence LN580135 from decomposed vegetation (Panama). The other five related environmental sequences within the ES1 clade also originated from forest soil habitats.

##### ES2

ASV 886 from Dikoe Lake, Urashnoe Lake, Malyj Kuchum Lake, Ugryumovo Lake, Berdyuzhe Lake, Bezrybnoe Lake, and Bolshoi Kurtal Lake (26–1682 mg/L) is grouped with full support with the environmental sequence KJ925320 from the Columbia River Estuary (USA). The environmental sequence AB572105 obtained from clay wall material (Japan) forms a sister lineage (91/1) to the abovementioned grouping.

##### ES3

ASV 2237 from Ushakovo Lake (145 mg/L) is grouped with full support within the ES3 clade with the environmental sequences LC150099 from domestic sewage waters (Japan) and KT272639 from forest litter and soil (Japan).

### Order Trivalvularida

#### Trivalvulariida

ASV 2284 from Dikoe Lake (145 mg/L) and ASV 1034 from Singul Lake (334 mg/L) are grouped together with full support. Marine benthic environmental sequence AY620334 (Portsmouth area coast, UK) forms a sister lineage to this group (50/0.75). The sister clade (99/1) for the abovementioned ASVs and the environmental sequence consists of marine benthic environmental sequences AY620333 and AY620335 (Portsmouth area coast, UK) and brackish water testate amoeba *Leptogromia operculate* Valkanov, 1970, sequences (MK325227, MK325225, and MK325226) from the Netherlands.

##### ES4

ASV 939 from Solyonoe Lake (11‰) clustered with high support (92/0.99) within the ES4 clade, together with planktonic marine environmental sequences HM369717 (USA), AY620338 (UK), HQ867335 (Canada), and HQ867234 (Canada). Freshwater sequence FJ353840 from the Industrial Canal (USA) forms a sister lineage to this grouping.

Figure 7 illustrates the grouping of 11 revealed ASVs among the order Cryomonadida (Rhogostomidae) and the order Tectofilosida (Fiscullidae, Pseudodifflugiidae, Rhizaspididae, and environmental clade ES5).

### Order Cryomonadida

#### Rhogostomidae

ASV 1897 from Travnoe Lake (444 mg/L) clusters (0.75/60) in a polytomy with sequences of *Rhogostoma minus* (LC032468 from the Czech Republic and AJ514867 from Russia) and three other sequences annotated as *Rhogostoma* sp. The BLAST similarity of ASV 1897 to these sequences is 99.8%. The abovementioned grouping forms a fully supported clade with *R. minus* (MN987026), *Rhogostoma micra* (HQ121436), and some environmental sequences.

ASV 1635 from Urashnoe Lake, Ushakovo Lake, Terenkul Lake, Travnoe Lake, and Bezrybnoe Lake (145–1,682 mg/L) is probably related to the plant/soil-associated clade (0.97/88) uniting sequences of *Rhogostoma epiphylla* Flues, Hermanns, et Dumack, 2017 (MN860280 and KY905095), *Rhogostoma medica* Öztoprak, 2020 (MN860285), and *Rhogostoma kyoshii* Öztoprak, 2020 (MN860287).

ASV 2082 from Chertankul Lake, Kalgan Lake, Terenkul Lake, Berdyuzhe Lake, and Bezrybnoe Lake (18–1,790 mg/L) occupies an unresolved position within Rhogostomidae.

ASV 1251 from Svetloe Lake, Singul Lake, Ugryumovo Lake, and Bolshoi Kurtal Lake (143–719 mg/L) is grouped with full support in an unresolved trichotomy with freshwater sequences from the Gerber Lakes, Central Pyrenees, Spain (HE655198) and Superior Lakes, USA (MW139907). The sister (1/98) fully supported clade to this grouping is formed by ASV 1323 from Svetloe Lake, Kalgan Lake, and Bolshoi Kurtal Lake (143–719 mg/L) and a marine environmental sequence from Portsmouth, UK (AY620276).

ASV 2112 from Solyonoe Lake (11‰) is grouped with an environmental sequence (FN690411) from Sweden sea ice (0‰–6‰) with high support within Rhogostomidae.

### Order Tectofilosida

#### Fiscullidae

ASV 3013 from Kalgan Lake and Bezrybnoe Lake (312–1,682 mg/L) clusters with high support (1/97) with sequences of *Fisculla siemensmai* Baumann, Dumack et Bonkowski, 2016 (KT809360) from a freshwater pond and *Fisculla asini* Baumann, Dumack, et Bonkowski, 2016 (KT809357) from the freshwater puddle.

##### ES5

ASV 472 from Chertankul Lake (18 mg/L) is grouped with the freshwater environmental sequence JN090862 (Greece), forming a sister lineage to a clade uniting all Fiscullidae and environmental sequences (GU479953 and AY620275) from Switzerland and the UK.

### Pseudodifflugiidae

ASV 1947 from Urashnoe Lake (147 mg/L) is grouped in an unresolved trichotomy with low support (0.63/54) with the sequence of *Pseudodifflugia* cf. *gracilis* Schlumberger, 1845 (AJ418794), from a freshwater biotope in Germany and freshwater environmental sequence AJ130858 from the Netherlands. The BLAST similarity of ASV 1947 to these sequences is 99.4%–99.6%, suggesting that this ASV may correspond to *Pseudodifflugia* cf. *gracilis*.

#### ES6

ASV 1590 from Travnoe Lake and Malyi Kuchum Lake (26–444 mg/L) and ASV 1385 from Singul Lake (334 mg/L) are grouped with each other with high support (0.99/97) and are sisters to Pseudodifflugiidae and Rhizaspididae.

### Testate amoebae distribution

**Fig 8:**
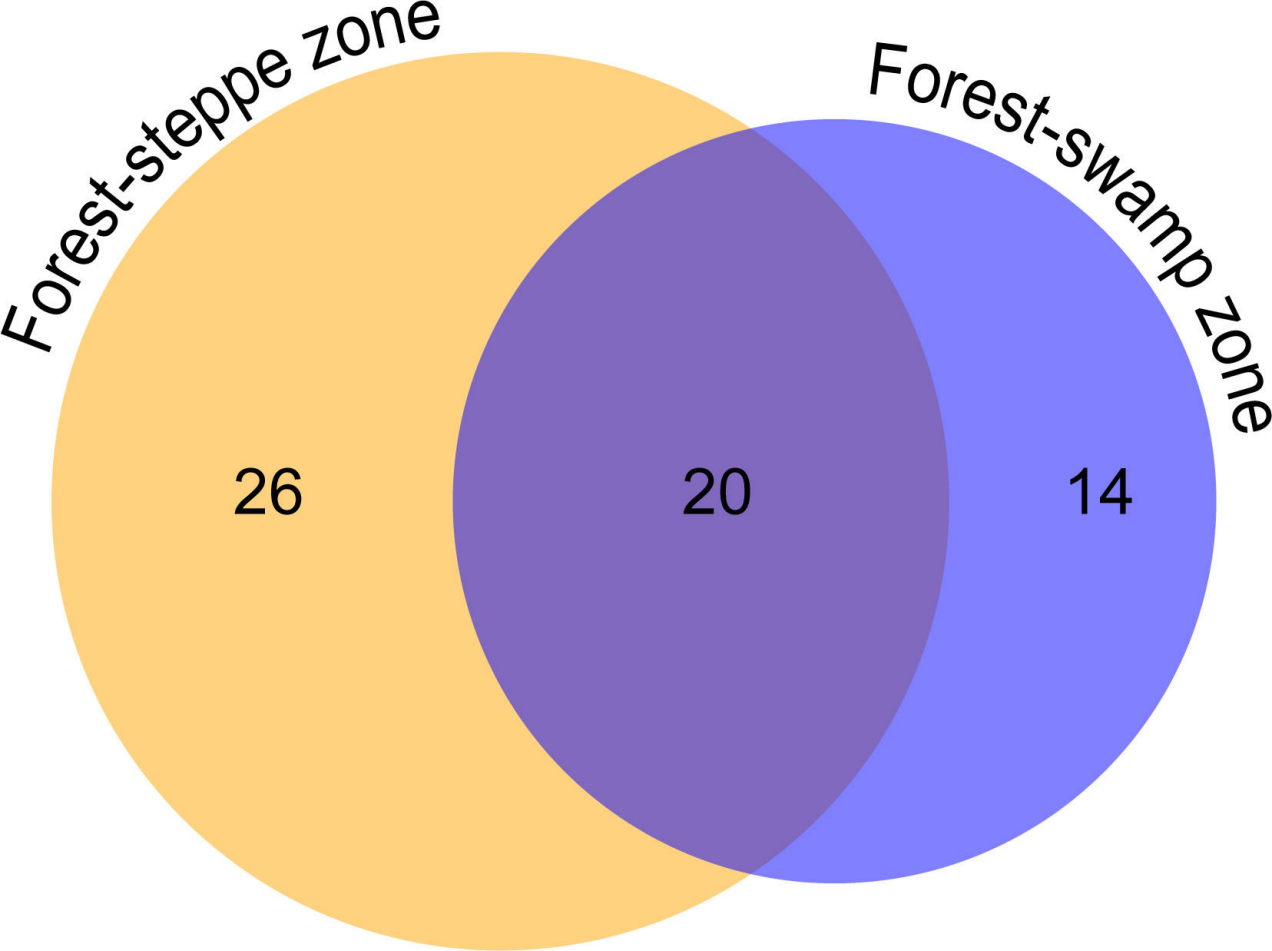
Venn diagram demonstrating the number of common and unique species and ASVs of testate amoebae in lakes of forest–swamp and forest–steppe zones.

In general, 34 species and ASVs of planktonic testate amoebae were identified using microscopy and metabarcoding for the biotopes of the forest–swamp zone, and 46 were identified for the forest–steppe zone. Twenty species and ASVs were common to the lakes of both natural zones (Fig. 8).

**Fig 9:**
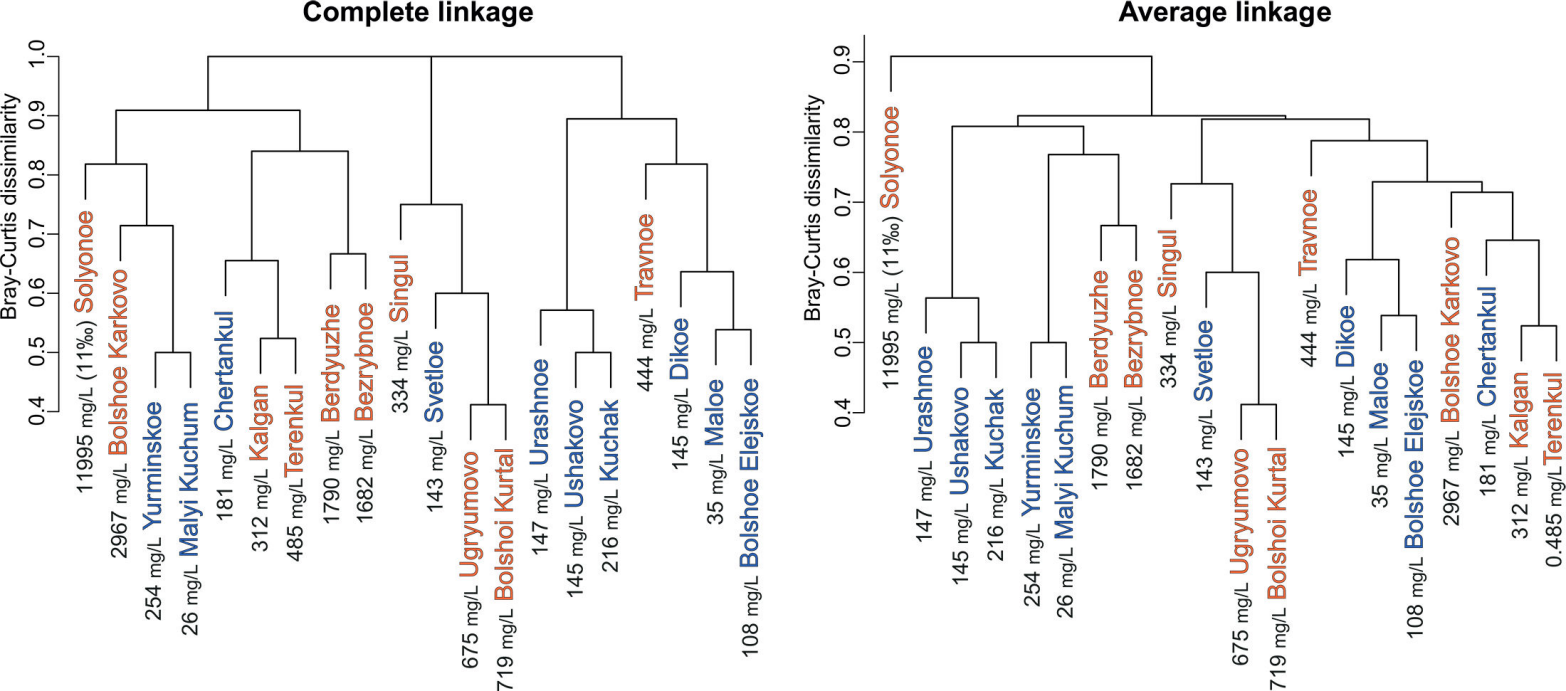
Cluster analysis (Bray–Curtis dissimilarity, complete and average linkage) based on the presence/absence of species and ASVs in the studied lakes. The forest–swamp zone lakes are marked in blue, and the forest–steppe zone lakes are marked in orange. Mineralization is indicated for each lake.

Classification of communities of planktonic testate amoebae by species and ASV composition using cluster analysis based on Bray–Curtis dissimilarity measure and average linkage and full linkage clustering algorithms did not reveal a clear grouping of lakes according to their location in natural zones (Fig. 9). However, average linkage clustering clearly showed a separation of the most mineralized and saline lake (Lake Solenoye, 11‰) from the other lakes in terms of species composition. Both clustering algorithms showed similarity in the species composition of communities of lakes with similar mineralization and salinity levels: Berdyuzhe Lake and Bezrybnoe Lake, with mineralization levels of 1,790 and 1,682 mg/L, respectively, and salinities of 1.43‰ and 1.34‰, respectively, and Ugryumovo Lake and Bolshoi Kurtal Lake, with mineralization levels of 675 and 719 mg/L, respectively, and salinities of 0.51‰ and 0.55‰.

**Fig 10:**
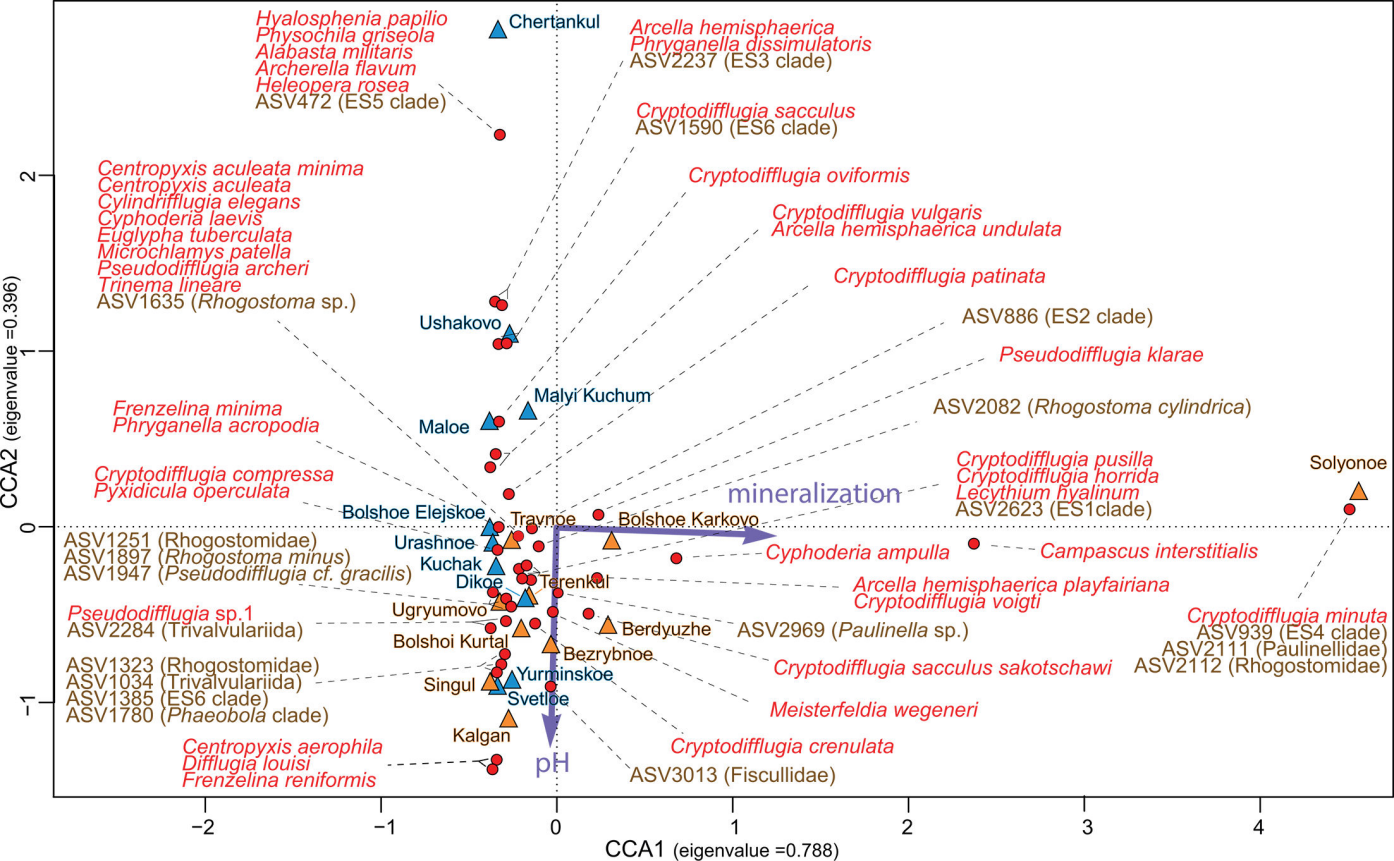
Canonical correspondence analysis (CCA) of testate amoebae distribution in the forest–swamp (blue) and forest–steppe (orange) lakes of Western Siberia.

The results of the canonical correlation analysis revealed that hydrological variables such as mineralization and pH corresponded to the CCA1 axis (eigenvalue = 0.788) and the CCA2 axis (eigenvalue = 0.396), respectively. At the same time, these variables explained only 22% of the variance. Moreover, only mineralization was a statistically significant parameter (Pr(>*F*) = 0.002) (Fig. 10). Forest–swamp lake testate amoebae communities were predominantly distributed along the CCA2 axis, while forest–steppe ones were predominantly distributed along the CCA1 axis.

Lake Solenoye was the most different from the other waterbodies and was characterized by a specific species composition of testate amoebae apparently associated with high mineralization. The occurrence and distribution of the species *Cyphoderia ampulla*, *Campascus interstitialis*, *Cryptodifflugia minuta*, ASV939 (ES4 clade), ASV2111 (Paulineliddae), and ASV2112 (Rhogostomidae) appeared to be related to mineralization. The occurrence and distribution of *Meisterfeldia wegeneri*, *Cryptodifflugia crenulate*, ASV2082 (*Rhogostoma cylindrica*), ASV2969 (*Paulinela* sp.), and ASV3013 (Fiscullidae) appeared to be related to pH.

## DISCUSSION

### Comparison of microscopy and metabarcoding methods and limitations of the study

Forty species from 20 genera of planktonic testate amoebae belonging to the Rhizaria and Amoebozoa supergroups were revealed using microscopy. Planktonic testaceans, ranging from 2 to 13 species per lake, were found in all lakes. At the same time, V4 18S rRNA metabarcoding revealed 20 ASVs belonging only to Rhizaria. Testate amoeba ASVs were found in 15 out of 20 lakes studied, from two to five ASVs per lake.

The taxonomic resolution of the two methods used was also different. Only 25% of the ASVs were classified down to the genus level (40% and 35% of the ASVs were classified to the order and family levels, respectively), whereas microscopic examination made it possible to identify 98% of the found shells to the species level and 2% to the genus level. At the same time, cryptic diversity within the morphospecies of testate amoebae has been demonstrated (51, 52), and in some cases, test shape appears to be more related to environmental characteristics than to the species’ phylogenetic position (29), which carries potential bias in taxonomic assignments on the bases of morphological data.

The testate amoebae genus *Pseudodifflugia* was revealed using both methods. Testate amoebae of the genera *Rhogostoma*, *Fisculla*, and *Paulinella* (Cercozoa and Rhizaria) were identified using only metabarcoding. In general, microorganisms belonging to Cercozoa are rarely reported in microscopic studies because they are difficult to identify (13), and the metabarcoding method is more effective in identifying the taxonomic composition of these ecologically important protists. Light microscopy often does not allow the identification of cercozoan testate amoebae (including *Rhogostoma* spp. and *Fisculla* spp.) due to the poor preservation of the organic covers of their shells and their small size (up to 8 µm) (15, 53).

Our results showed that general eukaryotic primers targeting the V4 18S rRNA gene are not suitable for identifying testate amoebae of the Amoebozoa supergroup, for which the use of primers targeting the cytochrome oxidase (COI) gene is more effective (52, 54, 55), whereas selected primers are well suited for identifying cercozoan testate amoebae. However, the relatively low number of ASVs obtained in this study may be due not only to primer and sampling biases but also to the lack of molecular data for most testate amoebae species, as well as the incompleteness and poor annotation of Cercozoa in databases. For example, more than 20 species are currently known for *Euglypha* spp., of which only nine are sequenced (18S rRNA gene) and annotated (in NCBI); for *Sphenoderia* spp., 21 species are known, but only five are sequenced and annotated; for *Trinema* spp., 8 species are known, of which two are sequenced and annotated; for *Corythion* spp., 5 species are known and one species is sequenced and annotated; for *Assulina* spp., 2 species are sequenced and annotated out of eight species, and one species of *Pseudodifflugia* spp. is sequenced and annotated out of the nine known species. There are no sequenced and annotated species for the genera *Campascus* Leidy, 1879; *Valkanovia* Tappan, 1966; *Schaudinnula* Awerintzew, 1907; *Puytoracia* Bonnet, 1970; *Playfairina* Thomas, 1961; *Deharvengia* Bonnet, 1979; Bobrov, Shimano, and Mazei, 2012; and *Pareuglypha* Penard, 1902. To improve the resolution of studies targeting specific taxonomic groups and habitat types, establishing specific metabarcoding protocols is desirable. Despite the nuances associated with the lack of molecular data for many taxonomic groups, the peculiarities of sample preparation, and the performance of primers, we clearly demonstrated that the universal eukaryotic primers used in this metabarcoding study are effective for the detection of pathogenic species of testate amoebae. The use of only one pair of universal primers is a significant advantage for carrying out high-quality environmental monitoring using metabarcoding, which is a rather expensive study.

The use of both microscopic and metabarcoding approaches provided complementary data on the diversity of planktonic testate amoebae, especially considering the new species described here. Therefore, both methods should be used for a more complete understanding of the taxonomic composition. The phylogenetic clades of environmental sequences revealed in this study highlight the existence of a hidden diversity of testaceans, the lack of exploration of which creates problems in interpreting the biodiversity and evolutionary relationships of testate amoebae. From an ecological perspective, we should note that assessing the abundance and biomass of testate amoebae using microscopy is very labor intensive and time consuming. At the same time, the metabarcoding approach revealed not only the taxonomic composition of testate amoebae but also the number of sequence reads for each taxon, which, according to some studies (13, 56), can be comparable to the biomass of the taxon, based on the assumption that a larger cell will contain more copies of the 18S rRNA gene. On the basis of the results of our study, the highest relative biomass among all planktonic testate amoebae was detected for ASV 472 (ES5, Tectofilosida), 25%; ASV 886 (ES2, Euglyphida), 11%; and ASV 939 (ES4, Trivalvularida), 10%. However, these estimates should be treated with caution because they are difficult to verify.

Considering the possible bias of the sampling method in this study, it should be noted that there is currently no consensus on whether planktonic testate amoebae are benthic species with a planktonic phase in their life cycle. It was reported that the Asian endemic *Netzelia tuberspinifera* (basionym *Difflugia tuberspinifera*) was found both in the plankton and in the benthos of the waterbody and that *N. tuberspinifera* sometimes leads a pelagic lifestyle (57), with the stimulus to leave the bottom being an abundance of food in the water column rather than temperature, and vertical movements in the water column for this protist are linked to circadian rhythms (58). In other studies, *N. tuberspinifera* was recorded in planktonic and periphytic environments with a high frequency, but it was very rarely or not observed in the benthos (59). We collected benthic samples from all sampling sites in Western Siberia lakes to identify testate amoebae species common to both plankton and benthos. In total, we identified 78 benthic species of testate amoebae, 19 species of which occurred in both plankton and benthos (Table 4).

**TABLE 4:** Planktonic and benthic testate amoebae of lakes in Western Siberia

Category	Organism
Exclusively planktonic testate amoebae	
1.	*Alabasta militaris*
2.	*Centropyxis aculeata minima*
3.	*Cryptodifflugia compressa*
4.	*Cryptodifflugia horrida*
5.	*Cryptodifflugia minuta*
6.	*Cryptodifflugia pusilla*
7.	*Cryptodifflugia sacculus*
8.	*Cryptodifflugia sacculus*
9.	*Cryptodifflugia vulgaris*
10.	*Cyphoderia ampulla*
11.	*Cyphoderia laevis*
12.	*Difflugia louisi*
13.	*Frenzelina minima*
14.	*Frenzelina reniformis*
15.	*Lecythium hyalinum*
16.	*Meisterfeldia wegeneri*
17.	*Phryganella dissimulatoris*
18.	*Physochila griseola*
19.	*Pseudodifflugia simensmai* sp. nov.
20.	*Pseudodifflugia klarae*
Testate amoebae common to both plankton and benthos	
21.	*Arcella hemisphaerica*
22.	*Arcella hemisphaerica playfairiana*
23.	*Arcella hemisphaerica undulata*
24.	*Archerella flavum*
25.	*Campascus interstitialis*
26.	*Centropyxis aculeata*
27.	*Centropyxis aerophila*
28.	*Cryptodifflugia crenulata*
29.	*Cryptodifflugia oviformis*
30.	*Cryptodifflugia patinata*
31.	*Cryptodifflugia voigti*
32.	*Cylindrifflugia elegans*
33.	*Euglypha tuberculata*
34.	*Heleopera rosea*
35.	*Hyalosphenia papilio*
36.	*Microchlamys patella*
37.	*Phryganella acropodia*
38.	*Pyxidicula operculata*
39.	*Trinema lineare*
Exclusively benthic testate amoebae	
40.	*Arcella costata* Ehrenberg, 1847
41.	*Arcella rotundata* Playfair, 1918
42.	*Arcella vulgaris* Ehrenberg, 1830
43.	*Assulina muscorum* Greeff, 1888
44.	*Centropyxis* sp.
45.	*Centropyxis aculeata lata* Decloitre, 1951
46.	*Centropyxis cassis* (Wallich, 1864), Deflandre 1929
47.	*Centropyxis delicatula* Penard, 1902
48.	*Centropyxis discoides* (Penard, 1902) Deflandre, 1929
49.	*Centropyxis ecornis* (Ehrenberg, 1841) Leidy, 1879.
50.	*Centropyxis elongata* (Penard, 1890) Thomas, 1959
51.	*Centropyxis minuta* Deflandre, 1929
52.	*Centropyxis orbicularis* Deflandre, 1929
53.	*Centropyxis platystoma* (Penard, 1890) Deflandre, 1929
54.	*Centropyxis sylvatica* (Deflandre, 1929) Bonnet and Thomas, 1955
55.	*Conicocassis pontigulasiformis* (Beyens et al., 1986) Nasser and Anderson, 2015
56.	*Cyclopyxis kahli* Deflandre, 1929
57.	*Cylindrifflugia acuminata* (Ehrenberg, 1838) González-Miguéns et al., 2022
58.	*Cylindrifflugia bacillariarum* (Perty, 1849) González-Miguéns et al., 2022
59.	*Cylindrifflugia lanceolata* (Penard, 1890) González-Miguéns et al., 2022
60.	*Difflugia ampulula* Playfair, 1918
61.	*Difflugia angustata* Deflandre, 1926
62.	*Difflugia bacillifera* Penard, 1890
63.	*Difflugia bryophila* (Penard, 1902) Jung, 1942
64.	*Difflugia fallax* Penard, 1890
65.	*Difflugia lacustris* (Penard, 1899) Ogden, 1983
66.	*Heleopera lucida* (Penard, 1890) Porfirio-Sousa et al., 2023
67.	*Difflugia minuta* Rampi, 1950
68.	*Difflugia minuta minor* Godeanu, 1972
69.	*Difflugia ovalisina* Chardez and Beyens 1994
70.	*Difflugia penardi* Cash and Hopkinson, 1909
71.	*Difflugia pristis* Penard, 1902
72.	*Difflugia sladeceki* Stepanek, 1967
73.	*Euglypha acanthophora* (Ehrenberg, 1841) Perty, 1849
74.	*Euglypha bryophila* Brown, 1911
75.	*Euglypha cabrolae* De Smet and Gibson, 2009
76.	*Galeripora arenaria* (Greeff, 1866) González-Miguéns et al., 2021
77.	*Galeripora catinus* (Penard, 1890) González-Miguéns et al., 2021
78.	*Galeripora discoides* (Ehrenberg, 1871) González-Miguéns et al., 2021
79.	*Galeripora megastoma* Penard, 1902
80.	*Geoplagiopyxis declivus* Chardez, 1960
81.	*Hyalosphenia elegans* Leidy, 1874
82.	*Lagenodifflugia epiouxi* (Chardez and Gaspar, 1984) Ogden, 1987
83.	*Lesquereusia spiralis* (Ehrenberg, 1840) Bütschli, 1888
84.	*Nadinella tenella* Penard, 1900
85.	*Nebela guttata* Kosakyan and Lara, 2012
86.	*Netzelia corona* (Wallich, 1864) Gooma et al., 2017
87.	*Netzelia gramen* (Penard, 1902) Gomaa et al., 2017
88.	*Netzelia tuberculata* (Wallich, 1864) Netzel, 1983.
89.	*Phryganella laurentiana* Nicholls, 2007
90.	*Plagiopxis navicula* (Bonnet, 1979) Meisterfeld, 2008
91.	*Plagiopyxis* sp.
92.	*Pseudodifflugia microstoma* Playfair, 1917
93.	*Pyxidicula cymbalum* Penard, 1902
94.	*Pyxidicula patens* Claparède and Lachman, 1858
95.	*Schwabia* sp.
96.	*Tracheleuglypha acolla* Bonnet and Thomas, 1955
97.	*Trinema enchelys* (Ehrenberg, 1838) Leidy, 1878

The planktonic testate amoebae identified through microscopy from unfiltered samples were less than 100 µm in size, with the exception of *Hyalosphenia papilio* Leidy, 1874. Only one exclusively planktonic species, *Cyphoderia ampulla* (Ehrenberg, 1840) Leidy, 1878, can potentially have a shell length of up to 124 µm, with an average of 106.5 µm (60). However, in our study, the size of *C. ampulla* reached 95 µm. Additionally, the species common to both the plankton and the benthos (Table 4) mostly had sizes up to 100 µm, and only for three species: *Centropyxis aculeata* (Ehrenberg, 1838) Stein, 1859; *Heleopera rosea* Penard, 1890; and *Hyalosphenia papilio* Leidy, 1874. The literature reports that their size exceeds 100 µm. In our study, the sizes of *C. aculeata* and *H. rosea* were both less than 100 µm. The shell size exceeded 100 µm only for *H. papilio*. However, even in this case, the testate amoeba can pass through the filter since it has a width of up to 60 µm. Therefore, the use of a 100 µm mesh filter in our study could not distort the results. Moreover, the three larger species are not typical planktonic testate amoebae but are associated with periphyton (59) and *Sphagnum* mosses (4, 61–63).

### Diversity of planktonic testate amoebae in different climatic zones of Siberia and biosafety threats

The lakes in the study area varied in terms of mineralization and included fresh and brackish waterbodies. The mineralization of lake waters increased from north to south as the climate became more continental and arid. Additionally, high mineralization is directly related to the recharge of such lakes with saline groundwater (64).

In the plankton of lakes moving from north to south, euryhaline species, such as *Campascus interstitialis*, which lives at water salinities ranging from 3.4‰ to 35.6‰ (65, 66), have appeared. Two representatives of the family Paulinellidae (according to the metabarcoding data) appear in forest–steppe lakes with salinities ranging from 0.55‰ to 11‰. At the same time, sphagnobiont testate amoebae, typical of boggy habitats and present in the studied lakes of the forest–swamp zone, disappeared in the lakes of the forest–steppe zone. The frequency of detection of planktonic testate amoeba ASVs was 1.5 times higher in forest–steppe lakes than in forest–swamp lakes. Lake Solenoe (11‰), located in the forest–steppe region, stood out among the other investigated lakes in terms of species composition and was distinctly segregated in both the cluster and CCA analyses. Four out of the six planktonic testate amoebae species detected here were recorded only in this lake. Notably, the *Cryptodifflugia minuta* found in Lake Solenoe was noted earlier as a characteristic species for habitats with an arid climate (67, 68). Testate amoeba ASVs 939 (ES4), 2111 (Paulinellidae), and 2112 (Rhogostomidae) were exclusively found in Solenoe Lake.

On the basis of microscopy data, moving from north to south (from the forest–swamp zone to the forest–steppe zone), the number and occurrence of filose species of testate amoebae increased. A total of 11 species of filose testate amoebae were found in the studied lakes. Of these, only four species were present in the lakes of the forest–swamp zone (zero to two species per lake), which have organic shells (*Lecythium* spp.), agglutinated shells (*Pseudodifflugia* spp.), or membranous tests with rare mineral grains (*Frenzelina* spp.). All 11 species of filose testaceans were found in the plankton of lakes in the forest–steppe zone, ranging from two to five species per lake. Testate amoebae with silica shells (*Campascus* spp., *Cyphoderia* spp., *Euglypha* spp., and *Trinema* spp.) were found only in the plankton of forest–steppe lakes. The increase in the number of species and occurrence (by 2.2 times) of filose testate amoebae in plankton moving to the south is apparently associated with an increase in mineral elements in the lakes necessary for the construction of shells, namely, silicon for testate amoebae of the order Euglyphida.

Among all the testaceans identified in this study, the largest number of species was noted for the genus *Cryptodifflugia* (11 species), which may indicate the preference (the greatest adaptability among testate amoebae) of this genus for a planktonic lifestyle.

Testate amoebae of the genera *Rhogostoma* and *Fisculla* may contain endosymbiotic bacteria of the order Legionellales (69, 70), which are common human pathogens that cause severe pneumonia. Infection with *Legionella* under natural conditions occurs by the inhalation of aerosols in which the bacterium is present (53, 71). These bacteria use testate amoebae of the genera *Rhogostoma* and *Fisculla* as hosts and are able to live in them. At the same time, the host cell is a kind of “Trojan horse” and promotes better preservation of bacteria, allowing the latter to more effectively resist disinfection as well as better spread and to acquire resistance to biocides (72).

In addition, testate amoebae of the genus *Rhogostoma*, present in most of the lakes studied, are capable of causing gill plate hyperplasia, resulting in one of the most serious diseases in fish, nodular gill disease (73, 74). Nodular gill disease is a relatively new amoebic disease of freshwater salmonids affecting rainbow trout, arctic char, chinook salmon, and brown trout farmed worldwide. The mortality of fish can reach 30% in a 1 month period when they are infected with *Rhogostoma* spp. (75). Timely diagnosis is hampered by the difficulty of detecting these testate amoebae in natural samples by microscopy due to their small size (6.5–8.0 µm) and poor shell preservation (76).

In this study, we identified four ASVs of testate amoebae that can harbor pathogenic bacteria. In the lakes of the forest–steppe zone, all four ASV types belonging to the genera *Rhogostoma* and *Fisculla* were present, whereas in the lakes of the forest–swamp zone, only two ASVs of the genus *Rhogostoma* were found. Moreover, the frequency of ASV occurrence of the genera *Rhogostoma* and *Fisculla* in the forest–steppe lakes was two times greater, and the number of sequence reads and, consequently, biomass was 2.3 times greater compared to forest–steppe lakes. This fact may indicate a greater preference by pathogenic amoebae for habitats with warmer and drier climates.

With expected further climate warming (77–82), the salinity and mineralization of freshwater lakes may increase (83–87), and they may also become alkalized (87). These changes may affect the species composition of planktonic testate amoebae, leading to an increase in pathogen-bearing testaceans and testate amoebae capable of causing fish diseases.

### Conclusions

A fairly large planktonic testate amoebae diversity was revealed in 10 lakes of the forest–swamp natural zone and 10 lakes of the forest–steppe natural zone of Western Siberia. Forty species belonging to Amoebozoa, Cercozoa, and Stramenopiles from 20 genera and 16 families have been identified via light microscopy. The described new species of testaceans was characterized by specific shell dimensions, shape, and aperture size. A metabarcoding investigation using universal eukaryotic primers for the 18S rRNA gene revealed 20 ASVs of planktonic testate amoebae within Cercozoa. The high-throughput amplicon sequencing revealed planktonic testate amoebae in only 75% of the studied waterbodies. The use of general eukaryotic 18S ribosomal RNA gene primers does not allow us to obtain full information about the taxonomic composition of planktonic testate amoebae in metabarcoding studies since these primers are only suitable for identifying cercozoan testaceans. We also note that the low number of revealed ASVs of planktonic testate amoebae is due not only to the insensitivity of the primers but also to the incompleteness of the databases and the lack of reference sequences. A combination of different sets of ribosomal DNA and cytochrome oxidase primers, as well as a combination of microscopy and metabarcoding approaches, allows for the most complete assessment of the diversity of testate amoebae in plankton. At the same time, the metabarcoding approach is an effective tool for identifying pathogenic cercozoan testaceans that are important in fish farming and environmental monitoring. Parasitic species of testate amoebae of the genus *Rhogostoma* were identified exclusively using the metabarcoding approach.

Although the taxonomic compositions revealed by the two methods were significantly different, they showed important differences in the communities of testate amoebae in the forest–swamp and forest–steppe natural zones. A greater frequency of ASV detection was noted in forest–steppe lakes with increased mineralization. The most mineralized waterbody (Lake Solenoe, 11‰) stood out among the other investigated lakes and was characterized by a specific testate amoeba composition, which included euryhaline species and a characteristic species for habitats with a dry and warm climate. Testate amoebae, typical of *Sphagnum* mosses, were found only in the plankton of lakes in the forest–swamp natural zone. In the plankton of lakes, from north to south, the number and occurrence of species of filose testate amoebae increased and euryhaline species appeared. Our study showed that in lakes in the forest–steppe zone, pathogenic testate amoebae are found twice as often, and their biomass was more than twice as high as that in forest–swamp lakes. This fact should be taken into account in the context of further climate warming in northern regions due to possible biosafety threats.

## Data Availability

The unprocessed eukaryotic 18S rRNA sequencing data set has been deposited in the National Center for Biotechnology Information and is available under BioProject accession number PRJNA1245471.
